# The associations between psychological distress and health-related quality of life in patients with non-cardiac chest pain

**DOI:** 10.1186/s12955-020-01297-0

**Published:** 2020-03-12

**Authors:** Ghassan Mourad, Jenny Alwin, Tiny Jaarsma, Anna Strömberg, Peter Johansson

**Affiliations:** 1 Department of Health, Medicine and Caring Sciences, Linköping University, Linköping, Sweden; 2 Department of Cardiology in Linköping, and Department of Health, Medicine and Caring Sciences, Linköping University, Linköping, Sweden

**Keywords:** Cardiac disease, EQ-5D-5 L, HRQoL, Non-cardiac chest pain, Psychological distress

## Abstract

**Background:**

Recurrent chest pain episodes with no clear explanation may affect patients’ psychological wellbeing and health-related quality of life (HRQoL) negatively. Despite the fact that a significant amount of patients with non-cardiac chest pain (NCCP) might have a history of Cardiac Disease (CD), there is today a lack of knowledge on how CD influences the association between psychological wellbeing and HRQoL in patients with NCCP. Therefore, the aim of this study is to describe HRQoL in patients with NCCP, with or without history of CD, and to explore the association between HRQoL and cardiac anxiety, depressive symptoms, fear of body sensations and somatization.

**Methods:**

Five hundred fifty-two patients discharged with NCCP from four hospitals in Southeast Sweden completed the EQ-5D, Cardiac Anxiety Questionnaire, Patient Health Questionnaire-9, Body Sensations Questionnaire, and Patient Health Questionnaire-15.

**Results:**

Fifty precent reported at least moderate problems regarding pain/discomfort and 25% reported at least moderate problems in the HRQoL dimensions mobility, usual activities, and anxiety/depression. Patients with NCCP and history of CD reported significantly lower HRQoL (*p* ≤ 0.05) compared to patients with NCCP without CD. In the total study population, cardiac anxiety, depressive symptoms, and somatization had weak significant negative associations (beta = 0.187–0.284, *p* < 0.001) with HRQoL. In patients with history of CD, the association between depressive symptoms and HRQoL was moderate (beta = − 0.339, *p* < 0.001), compared to weak association in patients without CD (beta = − 0.193, p < 0.001). On the other hand, the association between cardiac anxiety and HRQoL was weak in both patients with history of CD (beta = − 0.156, *p* = 0.05), and in those without (beta = − 0.229, *p* < 0.001).

**Conclusions:**

Patients with NCCP, in particular those with history of CD, reported low levels of HRQoL, which was associated with psychological distress. This should be considered when developing psychological interventions aiming to improve HRQoL in patients with NCCP.

## Key points

- NCCP patients, especially those with a history of CD, report poor HRQoL
- NCCP patients report psychological distress (i.e. cardiac anxiety, depressive symptoms and somatization)
- HRQoL is strongly associated with psychological distress

## Background

Non-cardiac chest pain (NCCP) is common in clinical cardiology and patients seek healthcare frequently due to recurrent and persistent chest pain. In many patients, no cardiac causes for the chest pain are found and patients are discharged with no clear explanation for their chest pain [1–5]. NCCP has been associated with psychological distress; predominantly anxiety, depression and fear [6–12]. In addition, patients with NCCP experience similar or higher levels of psychological distress compared to cardiac patients [6, 13, 14]. Several studies have confirmed the negative impact psychological distress has on patients’ health related quality of life (HRQoL) [6, 10, 15, 16].

Experiencing NCCP also has negative impact on patients’ HRQoL and everyday life, including interruption of daily activities and absence from work [7, 17–19]. Compared to other patient groups with chronic pain, patients with NCCP have lower HRQoL [20]. This should be taken seriously since HRQoL is an important patient-reported health outcome that is predictive of other health outcomes such as healthcare use and costs [21], especially in patients with chronic conditions where cure is not always an option. Thus, Husser et.al [22] emphasize the importance of identifying psychological distress in patients with NCCP since these patients experience a reduced quality of life. Therefore, it is important to identify and intervene factors such as cardiac anxiety, depressive symptoms, fear of body sensations and somatization that can modify patients´ perceived HRQoL.

Patients with NCCP might have had Cardiac Disease (CD) earlier [5, 23, 24], and hence tend to link their symptoms to the heart. As these new chest pain episodes are not diagnosed as cardiac, this may lead to worries and insecurity regarding the origin of the symptoms, which in turn may impact their HRQoL negatively. Our hypothesis is therefore that NCCP patients with history of CD have poorer HRQoL with stronger association to psychological distress than NCCP patients with no history of CD.

The aim of this study was therefore to describe HRQoL in patients with NCCP, with or without history of CD, and to explore the independent associations between HRQoL and cardiac anxiety, depressive symptoms, fear of body sensations and somatization in these patients.

## Materials and methods

This study had a descriptive cross sectional design. The design has been described in a previously published study [11] using the same data describing the prevalence of cardiac anxiety, depressive symptoms and fear of body sensations, and their relations to number of healthcare visits. In this study HRQoL is introduced and described, and its association to cardiac anxiety, depressive symptoms, fear of body sensations and somatization is analyzed. Furthermore, comparisons are made between patients with NCCP with or without history of CD.

### Study participants

Out of 2271 patients who were discharged with NCCP diagnoses (i.e. International Classification of Diseases (ICD) 10-codes R07.2, precordial chest pain; R07.3, other chest pain; R07.4, chest pain unspecified; and Z03.4, observation for suspected myocardial infarction) from four hospitals in southeast Sweden, 552 patients who met the inclusion criteria were included in the present study. Inclusion criteria were being 18 years or older and seeking medical care due to chest pain. Of the1719 non-participants, 1093 did not respond or complete the questionnaires, 406 declined participation, 181 reported other causes for their chest pain or declined chest pain, and 39 could not be reached or died during mail-out.

### Procedures

A package containing study information, written informed consent form, questionnaires, and a pre-stamped envelope was sent by post to all patients within one month from discharge. Patients were able to contact the research team for more information or in case of questions, and they agreed on study participation by signing and returning the written informed consent form together with the completed questionnaires.

### Data collection and measurements

All data was self-reported. Data consisted of background variables, health complaints including history of CD, number of healthcare visits due to NCCP, HRQoL, and psychological distress (i.e. cardiac anxiety, depressive symptoms, fear of body sensations, and somatization).

#### Health-related quality of life

The EQ-5D-5 L (five levels) and EQ-VAS was used to measure HRQoL. The EQ-5D is a reliable and valid instrument (Cronbach’s α coefficient 0.83 in this study). The EQ-5D includes five dimensions of HRQoL: mobility, self-care, usual activities, pain/discomfort and anxiety/depression. Each dimension has five levels corresponding to: no problems, slight problems, moderate problems, severe problems and extreme problems/unable. In the present study, each dimension was studied and an index value was calculated. There is currently no Swedish value set available for the calculation of the EQ-5D-5 L index, therefore a “cross walk” procedure was used [25] that was provided by the EuroQol Group [26]. The cross walk means that EQ-5D-5 L data was translated to a EQ-5D-3 L value set from which an index was calculated. In the present study, the UK value set [27], was used for the cross walk. The EQ-VAS is a visual analogue scale ranging from 0 (worst health you can imagine) to 100 (best health you can imagine). The scores are divided by 100 to easier compare to other population data [28].

#### Cardiac anxiety

Cardiac anxiety (i.e. fear of cardiac-related stimuli and sensations) was measured with the Cardiac Anxiety Questionnaire (CAQ), consisting of 18 items and a score range between 0 and 72. The higher scores the greater cardiac anxiety. In addition to a total score for the whole scale, the CAQ consists of three subscales for fear, avoidance, and heart-focused attention. In this analysis, only the total score was used. The total scale has demonstrated adequate reliability and validity [29], with Cronbach’s α coefficient of 0.90 in the present study.

#### Depressive symptoms

Depressive symptoms were measured using the Patient Health Questionnaire-9 (PHQ-9). This questionnaire comprises 9 items with scores between 0 and 27. At a score ≥ 10, the PHQ-9 has a sensitivity and a specificity of 88%, and a positive likelihood ratio of 7.1 to detect major depression. The PHQ-9 has demonstrated high internal consistency [30] with Cronbach’s α coefficient of 0.87 in the present study.

#### Fear of body sensations

Fear of body sensations (e.g. palpitations, dizziness and sweating), was measured with the 17-item Body Sensations Questionnaire (BSQ). The scores range between 17 and 85 and the higher scores the greater fear of body sensations [31]. The BSQ is valid and reliable [11, 31, 32] and has a Cronbach’s α coefficient of 0.93 in the present study.

#### Somatization

Somatization (i.e. reporting somatic symptoms with no pathophysiological cause) was measured with the Patient Health Questionnaire-15 (PHQ-15). This 15-item questionnaire measures physical symptoms on a scale from 0 to 30. Scores ≥10 indicate at least moderate somatization severity [33, 34]. The PHQ-15 is valid and reliable [33] and has a Cronbach’s α coefficient of 0.85 in the present study.

### Statistical analysis

The IBM SPSS version 24.0 was used for data analysis. Numbers, percentages, mean values and standard deviations were calculated to describe background variables and EQ-5D levels. For comparison between participants with or without history of CD, categorical variables were tested with Chi-Square tests while continuous variables were analyzed with Student’s t-test or Mann-Whitney U-test, depending on distribution of normality. To determine the independent relationship between psychological distress and HRQoL (EQ-5D VAS), multiple linear regression analysis was used in the total group as well as for the two groups with or without history of CD. The independent variables were entered in the regression model. In model 1, background variables´ (age, sex, educational level, marital status, work status, number of healthcare visits, and multi-morbidity) association to HRQoL was analyzed. In model 2, the different psychological distress variables (cardiac anxiety, depressive symptoms, fear of body sensations, and somatization) were added to model 1. The size of the associations were determined as weak based on beta values of 0.10–0.30, moderate between 0.30–0.50, and strong above 0.50. To assess the significance of difference in the strength of associations between the psychological variables and HRQoL in patients with and without history of CD, Fisher r-to z transformation was used. Analysis of influential cases was performed using Cook’s distance, and no value greater than 1 was found (min 0.000, max 0.039). Variation Inflation Factor (VIF) was used to assess multi-collinearity, and no value above 10 was found (highest value 2.44) [35]. *P* < 0.05 was considered as significant.

## Results

### Study participants

Study participants were 64 ± 17 years old, mainly married/cohabiting and more than half were retired, and the gender distribution was balanced (Table 1). Study participants were significantly younger than those who declined participation (70 ± 17 years, *p* < 0.001). One third (*n* = 188) of the patients had history of CD (i.e. angina pectoris, myocardial infarction, and/or heart failure). Patients with history of CD were older (71 vs. 60 years), more often male (60 vs. 44%), had lower educational levels, and more comorbidities than patients without a history of CD (4.7 vs. 2.8).

**Table 1:** Demographic data of patients with non-cardiac chest pain

	All patients^†^ (*N* = 552)	No history of cardiac disease (*n* = 360)	History of cardiac disease (*n* = 188)
Age year (mean ± SD)^a^	63.8 ± 16.6	59.9 ± 16.9	71.1 ± 12.9
Sex n (%)^a^			
Males	271 (49)	157 (44)	112 (60)
Females	281 (51)	203 (56)	76 (40)
Marital status n (%)			
Married/cohabiting	370 (67)	251 (70)	117 (62)
Single	180 (33)	107 (30)	71 (38)
Educational level n (%)^a^			
Up to compulsory school	185 (34)	91 (25)	91 (48)
High school	216 (39)	156 (43)	60 (32)
University	150 (27)	112 (31)	37 (20)
Work status n (%)^a^			
Working	152 (28)	125 (35)	27 (14)
Retired	302 (55)	167 (46)	132 (70)
Sick-leave/disability pension	40 (7)	24 (7)	16 (9)
Other	58 (10)	43 (12)	13 (7)
Number of healthcare visits n (%)^a^			
≤ 1 visit per year	331 (60)	241 (67)	88 (47)
2–3 visits per year	145 (26)	88 (24)	55 (29)
> 3 visits per year	76 (14)	31 (9)	44 (24)
Number of conditions (mean ± SD)^a^	3.5 **±** 2.2	2.8 ± 1.8	4.7 ± 2.3
Smoking n (%)			
None/previous smokers	493 (89)	325 (90)	166 (88)
Smokers	59 (11)	35 (10)	22 (12)
Alcohol consumption n (%)^a^			
None	141 (26)	74 (21)	65 (35)
≤ 7 glasses/week	390 (71)	269 (75)	119 (63)
> 7 glasses/week	20 (4)	16 (4)	4 (2)
Exercise n (%)^a^			
< 1 h/vecka	232 (42)	127 (35)	104 (55)
1–3 h/vecka	179 (32)	125 (35)	52 (28)
> 3 h/vecka	140 (25)	107 (30)	32 (17)

^†^= data regarding history of cardiac disease missing for four patients

^a^significant differences between patients with and patients without history of cardiac disease, *P* ≤ 0.01

### Health-related quality of life

**Fig. 1:**
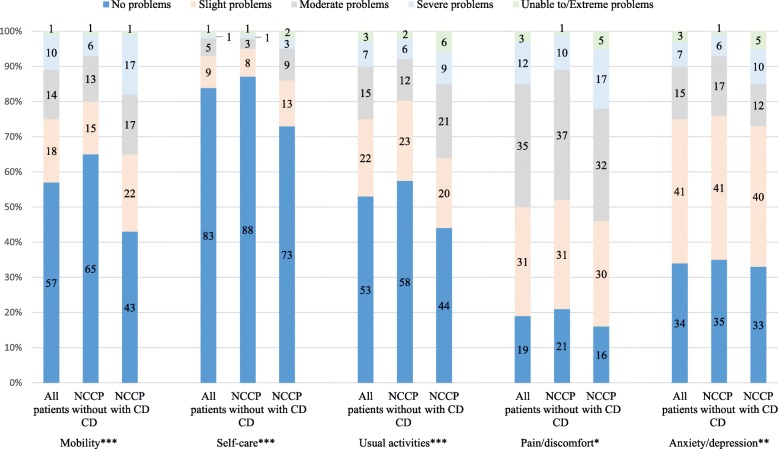
EQ-5D in all patients with non-cardiac chest pain (NCCP) (*N* = 552), and in NCCP without (*n* = 360) or NCCP with (*n* = 188) history of cardiac disease (CD). * = *p* < 0.05, ** = *p* < 0.01, *** = *p* < 0.001 regarding differences between the two groups

The scores for the EQ-5D VAS and the EQ-5D index for the total population were 0.7 ± 0.2 and 0.7 ± 0.3 respectively, Table 2. The five EQ-5D dimensions; mobility, self-care, usual activities, Pain/discomfort, and anxiety/depression are presented in Fig. 1. In total, 277 patients (50%) reported at least moderate problems regarding pain/discomfort, which was the dimension with most prevalent problems. About 25% (*n* = 136–139) of the patients reported at least moderate problems on the mobility, the usual activities, and the anxiety/depression dimensions. For self-care, 8% of the patients reported problems. Patients with history of CD scored significantly (*P* ≤ 0.05) higher on all dimensions indicating higher levels of problems, compared to patients with no history of CD.

**Table 2:** Health-related quality of life and psychological distress in patients with non-cardiac chest pain, (mean ± SD)

	All patients (*N* = 552)	No history of cardiac disease (*n* = 360)	History of cardiac disease (*n* = 188)	*P*-value
*Cardiac anxiety (CAQ)*^*‡*^	24.6 ± 13.0	22.7 **±** 11.8	28.2 ± 14.5	< 0.001
*Depressive symptoms (PHQ-9)*^*‡*^	6.4 ± 5.9	5.8 ± 5.4	7.4 ± 6.6	0.002
*Fear of body sensations (BSQ)*^*‡*^	31.4 ± 12.1	31.3 ± 11.7	31.5 ± 12.9	0.837
*Somatization (PHQ-15)*^*‡*^	10.0 ± 5.4	9.5 ± 5.2	10.8 ± 5.6	0.007
*Health-related quality of life (EQ-5D)*				
EQ-5D VAS	0.7 ± 0.2	0.7 ± 0.2	0.6 ± 0.2	< 0.01
EQ-5D index	0.7 ± 0.3	0.7 ± 0.2	0.6 ± 0.3	< 0.001

^*‡*^Data is partly presented in another article published in BMC Psychiatry. Permission to use these data will be requested from BMC Psychiatry

### Psychological distress

In mean, patients scored 24.6 ± 13 on the CAQ, 6.4 ± 5.9 on the PHQ-9, 31.4 ± 12.1 on the BSQ, and 10.0 ± 5.4 on the PHQ-15 (Table 2). Patients with history of CD scored significantly higher on all measurements, except from BSQ.

### The associations between psychological distress and HRQoL

Based on the total study population (Table 3), number of healthcare visits (beta = − 0.178, *p* < 0.001), multi-morbidity (beta = − 0.347, p < 0.001), older age (beta = 0.149, *p* = 0.001) and working (beta = 0.113, *p* = 0.015) were significantly associated with better HRQoL, see model 1 (R^2^ = 0.221, *p* < 0.001). When analyzing the groups (history of CD or not) separately, age and work status were not associated with HRQoL in patients with history of CD.

**Table 3:** The independent relationship between psychological distress and health-related quality of life shown by multiple linear regression analysis

Health-related quality of life, EQ-5D VAS						
	All patients (*N* = 552)^a^		No history of cardiac disease (*n* = 360)^b^		History of cardiac disease (*n* = 188)^c^	
Explanatory variables	Beta	*p*-value	Beta	*p*-value	Beta	*p*-value
Model 1						
Age	0.149	0.001	0.168	0.004	0.114	0.098
Sex	−0.007	0.854	0.025	0.611	−0.051	0.440
Education level	0.023	0.570	0.069	0.168	− 0.039	0.545
Marital status	−0.058	0.140	−0.030	0.541	−0.091	0.165
Work status	0.113	0.015	0.124	0.035	0.130	0.066
Number of healthcare visits	−0.178	< 0.001	−0.117	0.019	−0.272	< 0.001
Multi-morbidity	−0.347	< 0.001	−0.363	< 0.001	− 0.314	< 0.001
Model 2						
Age	0.030	0.451	0.085	0.106	−0.013	0.814
Sex	0.036	0.286	0.064	0.149	−0.008	0.887
Education level	−0.013	0.715	0.029	0.506	−0.066	0.217
Marital status	−0.033	0.322	0.016	0.710	−0.110	0.042
Work status	−0.068	0.088	0.102	0.049	0.071	0.223
Number of healthcare visits	−0.008	0.843	0.013	0.777	−0.002	0.976
Multi-morbidity	−0.056	0.173	−0.144	0.004	0.072	0.295
Cardiac anxiety	−0.187	< 0.001	−0.229	< 0.001	− 0.156	0.050
Depressive symptoms	−0.245	< 0.001	−0.193	0.001	−0.339	< 0.001
Fear of body sensations	0.000	0.994	0.056	0.278	−0.077	0.363
Somatization	−0.284	< 0.001	−0.258	< 0.001	− 0.320	< 0.001

Model 1: ^a^R^2^ = 0.221, F = 21.5, *p*-value< 0.001; ^b^R^2^ = 0.186, F = 11.3, *p*-value< 0.001; ^c^R^2^ = 0.302, F = 10.9, *p*-value< 0.001

Model 2: ^a^R^2^ = 0.434, F = 36.8, *p*-value< 0.001; ^b^R^2^ = 0.387, F = 19.6, *p*-value< 0.001; ^c^R^2^ = 0.557, F = 19.6, *p*-value< 0.001

In model 2 (R^2^ = 0.434, p < 0.001), after adding cardiac anxiety, depressive symptoms, fear of body sensations and somatization, none of the variables found in model 1 were significantly associated with HRQoL. Of the new variables added to the model, cardiac anxiety, depressive symptoms, and somatization had weak significant negative associations (beta = 0.187–0.284, *p* < 0.001) with HRQoL based on the total study population. There were differences in the final regression model between patients with or without history of CD. In patients with no history of CD, multi-morbidity was independently associated with HRQoL (beta = − 0.144, *p* = 0.004), but not in patients with history of CD (beta = 0.072, *p* = 0.295). Depressive symptoms had a moderate association with HRQoL in patients with history of CD (beta = − 0.339, *p* < 0.001), whereas this association was weak in patients with no history of CD (beta = − 0.193, p < 0.001) (Fisher r-to z transformation, *p* = 0.08). The associations between cardiac anxiety and HRQoL were weak in both patients with history of CD (beta = − 0.156, *p* = 0.05) and in those without (beta = − 0.229, p < 0.001), and did not differ significantly (Fisher r-to z transformation, *p* = 0.40). Furthermore, working (beta = 0.102, *p* = 0.049) was significantly associated with better HRQoL in patients with no history of CD, while being single (beta = − 0.110, *p* = 0.042) was associated with poorer HRQoL in patients with history of CD.

## Discussion

Non-cardiac chest pain is a common condition and has been found to be associated with high healthcare utilization rendering high healthcare and societal costs [12, 36]. Yet, few studies have previously described HRQoL in patients with NCCP. HRQoL is an important outcome in health economic analyses and this study can provide useful input in these analyses.

In the present study, we found that between 25 and 50% of those with NCCP had at least moderate problems on four of the five HRQoL dimensions (mobility, usual activities, pain/discomfort, and anxiety/depression). Four out of five of the total population reported at least slight problems with pain/discomfort and two third with anxiety/depression on the EQ-5D-5 L. These figures are higher compared to a general population in Sweden (*N* = 11,698, aged between 16 and 84 y), where 8–33% scored at least moderate problems on the same dimensions [37]. Patients with NCCP in this study reported poorer quality of life compared to a similar age group (60–69 years) in the general population in Sweden reflected by lower scores on both EQ-5D index and EQ-5D VAS (0.7 compared to 0.8 on the index, and 0.7 compared to 0.82 on the VAS) [28]. As hypothesized, patients with NCCP and history of CD have poorer HRQoL compared with NCCP patients with no history of CD, and they report the same level on the HRQoL index level (0.60) as ischemic heart disease patients in a previous study [37]. We also found that being single among those with CD was associated with poorer HRQoL. One possible explanation could be a need of more social support when living with a chronic disease, i.e. CD [38], but also that there is a higher likelihood of becoming depressed when having no partner [39], which in turn is related to poorer HRQoL.

Considering the fact that all study patients were discharged without planned follow-up, it is clear that these patients do not receive proper attention from the healthcare, in particular NCCP patients with history of CD, as CD itself has been found to be associated with poor HRQoL [40–42]. In addition, history of CD was found to be the only predictor of major adverse cardiac events in patients with NCCP [5]. In summary, patients with NCCP and in particular those with history of CD have poor HRQoL, which also was found in a recent study by Zhang et.al [43]. This suggests that actions must be taken to improve HRQoL in these patients.

We found that HRQoL was associated with cardiac anxiety, depressive symptoms and somatization, supporting previous results [44]. Both cardiac anxiety and depressive symptoms have been found to predict continued chest pain in NCCP patients [7, 15], thus leading to reduced HRQoL. As these patients often seek care due to recurrent and persistent chest pain [45–47], psychological mechanisms may serve as possible explanations for why patients continue to seek healthcare despite the fact that no physical cause can be determined. This suggests that cardiac anxiety and depressive symptoms are important to take into consideration in the treatment of patients with NCCP, when developing interventions to improve HRQoL. In NCCP patients with history of CD, depressive symptoms had a moderate negative association to HRQoL, whereas this association was weak in NCCP patients without CD, but this difference was not statistically significant (*p* = 0.08). Nor was there any significant difference between the groups regarding the association between cardiac anxiety and HRQoL, despite weak correlations, especially in patients without history of CD. Although psychological profile is similar between the groups [6, 13], NCCP patients with a history of CD might have depressive symptoms as a major response to their life situation, whereas NCCP patients without history of CD are more anxious as a response to the threat of developing CD [14, 48]. Previous research including a study based on the English General Practice Patient Survey 2011–2012 showed that multi-morbidity was associated with reduced quality of life [49, 50]. Unexpectedly, in patients with history of CD in our study, multi-morbidity was not associated to HRQoL, which was the case in those without history of CD. This finding is not easy to explain, but we hypothesize that NCCP patients with a history of CD in a higher extent focus their symptoms to their already confirmed cardiac diagnosis, compared to those without a history of CD. Consequently, this means that patients in the latter group are more likely to focus their symptoms not only to the heart but also to other diagnoses, which can further impact their HRQoL. Interventions aiming to decrease psychological distress may be used to improve patient outcomes and to teach patients with NCCP how to handle their chest pain, and thus improve HRQoL. Our findings (i.e. the differences between the groups) may suggest that these interventions should be tailored in relation to previous experience of CD. However, before development of such tailored interventions, more research is needed to explore if different psychological variables have different associations to HRQoL in NCCP depending on previous experience of CD.

### Limitations

The study team had no access to medical charts and therefore approached all possible patients based on few inclusion criteria. This may have led to low response rate and some differences in demographical profile between the responders and non-responders as younger patients did not respond at all and older patients declined participation.

## Conclusions

Patients with NCCP, in particular those with a history of CD, report lower levels of HRQoL as compared with the general population, and a high proportion of the patients experience problems with pain/discomfort and anxiety/depression. Cardiac anxiety, depressive symptoms and somatization are independently associated with HRQoL, but differ slightly with regard to history of CD. This highlights the diversity of the NCCP group and that different aspects of psychological distress may be influenced by previous experience of CD, which should be considered in the development of psychological interventions aiming to decrease psychological distress and improve HRQoL in patients with NCCP.

## Data Availability

The datasets used and/or analyzed during the current study are available from the corresponding author on reasonable request.

## Abbreviations

BSQ: Body Sensations Questionnaire
CAQ: Cardiac Anxiety Questionnaire
CD: Cardiac Disease
HRQoL: Health-related quality of life
ICD: International Classification of Diseases
NCCP: Non-Cardiac Chest Pain
PHQ-15: Patient Health Questionnaire-15
PHQ-9: Patient Health Questionnaire-9
VIF: Variation Inflation Factor
